# Indigenous art-themed personalised theatre caps improve patient perioperative experience and perceived staff communication in the operating theatre: a quality improvement project at Royal Darwin Hospital in Australia

**DOI:** 10.1186/s13104-024-06690-2

**Published:** 2024-01-21

**Authors:** Benjamin Peake, Alexander Smirk, Guy Debelak

**Affiliations:** Department of Anaesthesia and Perioperative Medicine, Royal Darwin Hospital, Darwin, NT Australia

**Keywords:** Communication, Patient experience, Quality improvement, Surgical safety, Teamwork, Indigenous, Anaesthesia, Theatre cap, Scrub cap, Cultural competence

## Abstract

**Background:**

Personalised theatre caps have been shown to improve staff communication in the operating theatre. The impact of these caps on the patient perioperative experience, particularly in Indigenous Australian patients, has not been well established.

**Methodology:**

Surgical patients and operating theatre staff at Royal Darwin Hospital in Australia were surveyed before and after the introduction of Indigenous art-themed personalised (name and role) theatre caps in October 2021 and January 2022. Staff name and role visibility in operating theatres was also audited.

**Results:**

A total of 223 staff and patients completed surveys. Most patients reported the theatre caps to be helpful (90%, 95% confidence interval [CI] 81–99) and felt more comfortable because staff were wearing them (91%, 95% CI 82–100). These results were consistent across Indigenous and non-Indigenous patients. The majority of staff agreed that personalised name and role theatre caps improved staff communication (89%, 95% CI 81–97), improved the staff-patient interaction (77%, 95% CI 67–87), and made it easier to use staff names (100%). Staff name and role visibility increased from 8 to 51% (*p* < 0.001) after the introduction of personalised theatre caps.

**Conclusions:**

The introduction of Indigenous art-themed personalised theatre caps for operating theatre staff at Royal Darwin Hospital improved perceived staff communication and the patient perioperative experience.

## Introduction

Personalised (name and role) theatre caps have been shown to improve staff communication in the operating theatre [1, 2]. Staff are more likely to know and use colleagues’ names if name and role caps are worn [1, 2]. It is estimated that up to 70% of adverse events in healthcare are due to communication errors [3], and difficulty with name recall can impede communication and increase the risk of error [4].

The impact of these caps on the patient perioperative experience, particularly in Indigenous Australian patients, has not been well established. The term “Indigenous” is used in this paper to encompass both Aboriginal and Torres Strait Islander people. Wearing personalised theatre caps made from Indigenous art-themed fabrics may assist delivery of culturally competent patient care [5] and such competence may improve health care experiences and outcomes [6].

Fifty percent of hospital admissions at Royal Darwin Hospital identify as Indigenou**s.** Royal Darwin Hospital is a 360-bed public hospital located in Darwin, Northern Territory, Australia, that provides approximately 12,000 episodes of surgical care per year. The theatre complex at Royal Darwin Hospital contains nine operating theatres and performs a wide range of adult and paediatric surgical services, excluding cardiac surgery.

The introduction of reusable theatre caps may also have environmental and economic benefits [7, 8].

The purpose of the study was to assess the acceptability and potential benefits of introducing personalised, Indigenous art-themed reusable theatre caps (including name and role) for staff in the operating theatre at Royal Darwin Hospital on staff communication and the patient perioperative experience.

## Methodology

The study was granted full ethical approval by the Human Research Ethics Committee of the Northern Territory Department of Health and Menzies School of Health Research, and Aboriginal Ethics Sub-Committee (Approval No. HREC-2021-4122). Informed consent was obtained from all participants. The Aboriginal Liaison service at Royal Darwin Hospital was consulted and supported the study.

Surgical patients and operating theatre staff were asked to complete a short online survey before and after the introduction of Indigenous art-themed personalised (name and role) theatre caps. The survey questions for staff and patients, before and after introduction of personalised theatre caps, are listed in Table 1.

**Table 1:** Survey questions

Patient questions	
Before introduction of personalized theatre caps	(Yes, No or Maybe)
– Do you think it’s a good idea for operating theatre staff to wear their name and job on their theatre hat?	
– Would you support the introduction of staff name and job theatre hats designed with indigenous art-themed fabrics?	
– Do you identify as Aboriginal or Torres Strait Islander?	(Yes, No, or Prefer not to answer)
After introduction of personalized theatre caps	(Yes, No or Maybe)
– Did you notice that staff were wearing their names and jobs on their hats today?	
– If yes, was this helpful?	
– If yes, did this make you feel more comfortable or less comfortable?	(More, Less or No difference)
– Do you identify as Aboriginal or Torres Strait Islander?	(Yes, No or Prefer not to answer)

Staff questions	
Before introduction of personalized theatre caps	(Yes, No or Maybe)
– Would you support the introduction of personalised (name and role) reusable theatre caps for operating theatre staff at Royal Darwin Hospital?	
– Would you support the introduction of personalised reusable theatre caps designed with indigenous art-themed fabrics?	
– Would you be willing to pay $25 for a personalised reusable theatre cap?	
After introduction of personalized theatre caps	(Agree, Disagree or Neither agree nor disagree)
Regarding the introduction of personalised (name and role) theatre caps in the operating theatre:	
– The theatre caps have improved staff communication	
– The theatre caps have improved the staff-patient interaction during the perioperative period	
– The theatre caps have made it easier to use staff names	
– The theatre caps have made it easier to raise concerns	
– I would support the ongoing use of personalised (name and role) theatre caps in the operating theatre	

Online surveys were completed before and after the introduction of personalised theatre caps, for 1 week in October 2021 and 1 week in January 2022. Staff members working in the operating theatre were approached when available (i.e., not scrubbed or involved in emergent clinical care) and invited to participate. This occurred throughout the survey periods to capture as many operating theatre staff as possible. All adult patients undergoing elective or emergency surgery were eligible. When English was not spoken as a first language, interpreters involved in routine patient care were asked to assist with completion of the questionnaire. Patients and staff could access the online surveys by scanning a quick response (QR) code with their mobile-device or by using a hospital-owned iPad. Participation was voluntary and informed consent was obtained as part of the online questionnaire.

**Fig. 1:**
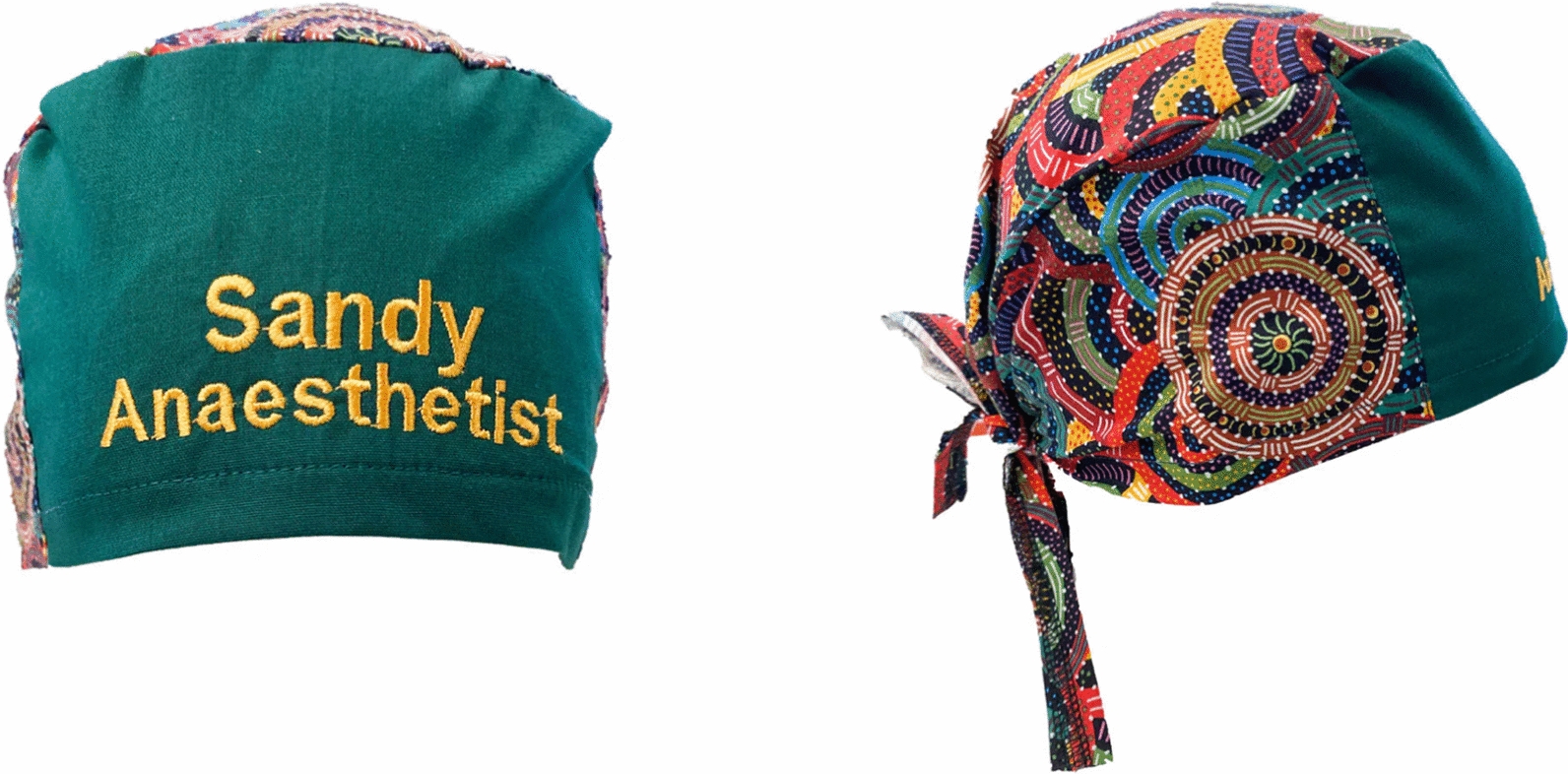
Example of personalised theatre cap

Following the staff and patient surveys in October 2021, Indigenous art-themed personalised (name and role) reusable theatre caps were made available to operating theatre staff for purchase through an online supplier of operating theatre clothing (Hunter Scrubs, New South Wales, Australia). Eleven Indigenous art-themed fabrics were available. The artist’s name and a story behind each artwork was provided. Staff name and role was embroidered on a solid-color panel at the front of the cap for ease of reading. The name was embroidered above the role and staff were free to decide on their name and role, without standardization. It was not compulsory for operating theatre staff to wear personalised theatre caps. Those that did were asked to wash their caps daily, in accordance with published guidelines [9]. An example of a personalised theatre cap is shown in Fig. 1.

Staff name and role visibility in operating theatres was audited on day one of each survey period. This was done by a person standing at the door of each operating theatre looking at staff from a distance.

Proportions and 95% confidence intervals were calculated for patient and staff survey responses.

## Results

A total of 223 staff and patients completed before and after surveys, which included 118 staff (53 before, 65 after) and 105 patients (51 before, 54 after). All staff invited to participate in the survey did so, regardless of whether they were wearing a personalised theatre cap. Most patients completed the survey when offered, however the exact patient response rate was not recorded. The number of patients who utilised an interpreter to help complete the survey was not recorded. Survey responses are shown in Tables 2 and 3.

**Table 2:** Patient responses to survey questions

Patient—Before	Overall (n = 51)			Indigenous (n = 9)		
	% (95% CI)			% (95% CI)		
Abbreviated survey question	Yes	Maybe	No	Yes	Maybe	No
Support personalised theatre caps?	86 (76–96)	12 (3–21)	2 (0–6)	78 (51–100)	11 (0–31)	11 (0–31)
Support indigenous-art themed personalised theatre caps?	78 (67–89)	18 (7–29)	4 (0–9)	78 (51–100)	11 (0–31)	11 (0–31)

Patient—After	Overall (n = 54)			Indigenous (n = 14)		
	% (95% CI)			% (95% CI)		
Abbreviated survey question	Yes	Maybe	No	Yes	Maybe	No
Noticed personalised theatre cap?	74 (62–86)	11 (3–19)	15 (5–25)	57 (31–83)	29 (5–53)	14 (0–32)
Helpful?	90 (81–99)	10 (2–18)	0	89 (69–100)	11 (0–27)	0
More comfortable?	91 (82–100)	–	9* (1–17)	88 (65–100)	–	12* (0–29)

*No = No difference

**Table 3:** Staff responses to survey questions

Staff—Before	Overall (n = 53)		
	% (95% CI)		
Abbreviated survey question	Yes	Maybe	No
Support personalised theatre caps?	94 (88–100)	2 (0–6)	4 (0–9)
Support indigenous-art themed personalised theatre caps?	91 (83–99)	8 (1–15)	2 (0–6)
Willing to pay $25?	77 (66–88)	15 (5–25)	8 (1–15)

Staff—After	Overall (n = 65)		
	% (95% CI)		
Abbreviated survey question	Yes	Neither agree nor disagree	No
Improved staff communication?	89 (81–97)	11 (3–19)	0
Improved staff-patient interaction?	77 (67–87)	23 (13–33)	0
Easier to use staff names?	100	0	0
Easier to raise concerns?	65 (53–77)	31 (20–42)	5 (0–10)
Support ongoing use of personalised theatre caps?	91 (84–98)	9 (2–16)	0

The proportion of staff wearing theatre caps with visible name and role increased from 8% (n = 5, out of 63) in October 2021 to 51% (n = 26, out of 51) in January 2022 (*p* < 0.001). Many staff groups, including anaesthetists, surgeons, nursing staff, theatre technicians, midwives, obstetricians, and paediatricians, wore personalised theatre caps.

Most patients (78%, 95% confidence interval [CI] 67–89) and staff (91%, 95% CI 83–99) were supportive of the introduction of Indigenous art-themed personalised theatre caps for operating theatre staff at Royal Darwin Hospital. After introduction of the caps, almost all staff (91%, 95% CI 84–98) were supportive of their ongoing use.

The proportion of patients who identified as Aboriginal or Torres Strait Islander was 18% in the “before” and 26% in the “after” patient survey. The majority of patients surveyed reported the personalised theatre caps to be helpful (90%, 95% CI 81–99) and felt more comfortable because staff were wearing them (91%, 95% CI 82–100). These results were consistent across Indigenous and non-Indigenous patients.

Most staff agreed that personalised name and role theatre caps improved staff communication (89%, 95% CI 81–97), improved the staff-patient interaction (77%, 95% CI 67–87), and made it easier to use staff names (100%). Many staff also agreed that personalised theatre caps made it easier to raise concerns (65%, 95% CI 53–77).

## Discussion

The introduction of Indigenous art-themed personalised theatre caps (name and role) for operating theatre staff at Royal Darwin Hospital improved perceived staff communication and the perioperative experience for Indigenous and non-Indigenous patients.

The survey results showing the positive impact of name and role caps on staff communication in the operating theatre was like that seen in previous studies [1, 2]. Slightly fewer staff (65%) at Royal Darwin Hospital agreed that the caps made it easier to raise concerns. This suggests that whilst knowing a colleague’s name and role is important in speaking up, other workplace factors also play a role. Hierarchy gradients, organisational culture, and education are frequently observed as factors affecting ability to challenge authority [10].

Indigenous and non-Indigenous patients who noticed the caps found them helpful and felt more comfortable because staff were wearing them. It is unclear whether the visible name and role or the Indigenous art-themed fabrics themselves were responsible for the improved perioperative experience in Indigenous patients who noticed the caps. A previous study at Royal Darwin Hospital demonstrated that only 17.7% of Aboriginal patients presenting to the operating theatre spoke English as their first language [11]. Future rollout of Indigenous-art themed personalised theatre caps could consider including names in both English and a local Indigenous language, however there are more than 100 Indigenous languages and dialects spoken in the Nothern Territory in Australia [12] and choosing one may only tailor to a minority of the Indigenous population.

There are several limitations associated with this study. Firstly, only half (51%) of operating theatre staff had their name and role visible after the personalised theatre caps were introduced. This may have underestimated any staff communication or patient experience benefit demonstrated in the study.

Secondly, most patients completed the survey when offered, however the exact patient response rate was not recorded. This could bias the results if these non-responders were more likely to either support or not support the caps. Perioperative time pressure, family or medical team presence, post-operative discomfort and interpreter availability were the reasons quoted for patient non-completion. Interpreters involved in routine care were utilised to complete surveys, but they were not always present at the bedside when surveys were being performed, particularly post-operatively. This may partly explain why the proportion of Indigenous patients who completed surveys (22%) was lower than the percentage of patients admitted to Royal Darwin Hospital who identify as Indigenous (50%).

Thirdly, operating theatre staff were only surveyed if they were not scrubbed or involved in emergent clinical care at the time. Surgeons and scrubs nurses, staff who are often scrubbed, may therefore have been underrepresented as survey respondents.

Fourthly, the sample size for staff, patients, and in particular Indigenous patients completing surveys was relatively small and may limit generalizability to a larger population. Moreover, apart from Indigenous-status, demographic data was not collected.

Finally, despite demonstrating an improvement in perceived staff communication, the study is unable to comment on whether personalised theatre caps reduce the incidence or improve the management of clinical crises.

## Conclusion

The introduction of Indigenous art-themed personalised (name and role) theatre caps for operating theatre staff at Royal Darwin Hospital has improved perceived staff communication and the perioperative experience for Indigenous and non-Indigenous patients. Further research is required to assess whether personalised theatre caps reduce the incidence or improve the management of clinical crises.

## Data Availability

Data and materials used in this study are available and can be presented by the corresponding author upon reasonable request.
